# Supplementary material for: The Systems Biology Research Tool: evolvable open-source software
Source: BMC Syst Biol. 2008 Jun 29;2:55. doi: 10.1186/1752-0509-2-55 (PMC2446383; doi:10.1186/1752-0509-2-55)
Supplement: Additional file 1 — SBRT Archive. An archive of the current version of the Systems Biology Research Tool. [file 1752-0509-2-55-S1.zip › sbrt-1.4.0/doc/developers_guide/api/sbrt/shell/io/fba/class-use/FbaOptOutputFile.html]

Uses of Interface sbrt.shell.io.fba.FbaOptOutputFile


|  |  |  |
| --- | --- | --- |
| **Overview** \| **Package** \| **Class** \| **Use** \| **Tree** \| **Deprecated** \| **Index** \| **Help** | |  |
| PREV   NEXT | **FRAMES**    **NO FRAMES**     **All Classes** |  |


---


## **Uses of Interface sbrt.shell.io.fba.FbaOptOutputFile**

| Packages that use FbaOptOutputFile | |
| --- | --- |
| **sbrt.shell.io.fba** | Provides classes and interfaces for reading and writing files relevant to Flux Balance Analysis. |
| **sbrt.shell.mng.fba** | Provides classes and interfaces for creating Flux Balance Analysis process managers. |

Uses of FbaOptOutputFile in sbrt.shell.io.fba

| Classes in sbrt.shell.io.fba that implement FbaOptOutputFile | |
| --- | --- |
| `class` | `ColumnOrFbaOptOutputFile`             This class is used to write column-oriented output files containing FBA optimization data. |
| `class` | `RowOrFbaOptOutputFile`             This class is used to write row-oriented output files containing FBA optimization data. |
| `class` | `SerFbaOptOutputFile`             This class is used to write output files containing FBA optimization data as serialized maps. |

Uses of FbaOptOutputFile in sbrt.shell.mng.fba

| Methods in sbrt.shell.mng.fba that return FbaOptOutputFile | |
| --- | --- |
| `static FbaOptOutputFile` | `FbaOptManager.getMultiResultsOutputFile(ManagerInput<java.lang.String,java.lang.String> input, java.io.PrintWriter out, Fluxome fluxome)`             Returns an empty output file that is suitable for storing multiple FBA optimization results. |
| `static FbaOptOutputFile` | `FbaOptManager.getMultiResultsOutputFile(java.lang.String fileName, FileFormat fileFormat, java.util.Set<java.lang.String> dataHeaders, Fluxome fluxome)`             Returns an empty output file that is suitable for storing multiple FBA optimization results. |
| `static FbaOptOutputFile` | `FbaOptManager.getSingleResultOutputFile(ManagerInput<java.lang.String,java.lang.String> input, java.io.PrintWriter out, Fluxome fluxome)`             Returns an empty output file that is suitable for storing a single FBA optimization result. |
| `static FbaOptOutputFile` | `FbaOptManager.getSingleResultOutputFile(java.lang.String fileName, FileFormat fileFormat, java.util.Set<java.lang.String> dataHeaders, Fluxome fluxome)`             Returns an empty output file that is suitable for storing a single FBA optimization result. |

| Methods in sbrt.shell.mng.fba that return types with arguments of type FbaOptOutputFile | |
| --- | --- |
| `static MultiOutputFiles<? extends FbaOptOutputFile,FbaOptComputer.Results>` | `FbaOptManager.getMultiFileOutput(ManagerInput<java.lang.String,java.lang.String> input, java.io.PrintWriter out, java.util.Iterator<java.lang.String> fileNameSource, java.lang.String fileNameSourceName, Fluxome fluxome)`             Please consult the source code for a complete description of this method's actions. |

---


|  |  |  |
| --- | --- | --- |
| **Overview** \| **Package** \| **Class** \| **Use** \| **Tree** \| **Deprecated** \| **Index** \| **Help** | |  |
| PREV   NEXT | **FRAMES**    **NO FRAMES**     **All Classes** |  |


---
